# Insight study on synthesis and antibacterial mechanism of silver nanoparticles prepared from indigenous plant source of Jharkhand

**DOI:** 10.1186/s43141-023-00463-3

**Published:** 2023-03-10

**Authors:** Koel Mukherjee, Namrata Bhagat, Madhubala Kumari, Arnab Roy Choudhury, Biplab Sarkar, Barnali Dasgupta Ghosh

**Affiliations:** 1 Department of Bioengineering and Biotechnology, Birla Institute of Technology, Mesra, Jharkhand 835215 Ranchi, India; 2 Downstream Agro-Processing Division, ICAR-National Institute of Secondary Agriculture, Namkum, Jharkhand 834010 Ranchi, India; 3 Indian Council of Agricultural Research-Indian Institute of Agricultural Biotechnology, Garhkhatanga, Jharkhand, 834010 Ranchi, India; 4 Present Address: Department of Chemistry, Birla Institute of Technology, Mesra, Ranchi, Jharkhand 835215 India

**Keywords:** Ag-NPs, Indigenous plant, *Litsea glutinosa*, *Polygonum plebeium*, *Vangueria spinosus*

## Abstract

**Background:**

The Ag-NPs by green synthesis has a notable interest because of their eco-friendliness, economic views, feasibility, and applications in a wide range. Herein, native plants of Jharkhand (*Polygonum plebeium*, *Litsea glutinosa*, and *Vangueria spinosus*) were selected for the current work of Ag-NP synthesis and further antibacterial activity. Green synthesis was performed for Ag-NPs using Silver nitrate solution as precursor and the dried leaf extract performs as a reductant and stabilizer here.

**Result:**

Visually Ag-NP formation was observed along with a colour change and confirmed by UV-visible spectrophotometry on which an absorbance peak occurs at around 400–450nm. Further characterization was done on DLS, FTIR, FESEM, and XRD. Size around 45–86 nm of synthesized Ag-NPs was predicted through DLS. The synthesized Ag-NPs exhibited significant antibacterial activity against *Bacillus subtilis* (Gram-positive bacteria) and *Salmonella typhi* (Gram-negative bacteria). The finest antibacterial activity was disclosed by the Ag-NPs synthesized by *Polygonum plebeium* extract. The diameter of the zone of inhibition in the bacterial plate measured was 0–1.8 mm in *Bacillus* and 0–2.2 mm in *Salmonella typhi*. Protein-Protein interaction study was performed to study the effect of Ag-NPs towards different antioxidant enzyme system of bacterial cell.

**Conclusion:**

Present work suggest the Ag-NPs synthesized from *P. plebeium* were more stable for long term and might have prolonged antibacterial activity. In the future, these Ag-NPs can be applied in various fields like antimicrobial research, wound healing, drug delivery, bio-sensing, tumour/cancer cell treatment, and detector (detect solar energy).

**Graphical abstract:**

Schematic representation of Ag-NPs green synthesis, characterization, antibacterial activity and at the end, in silico study to analyse the mechanism of antibacterial activity
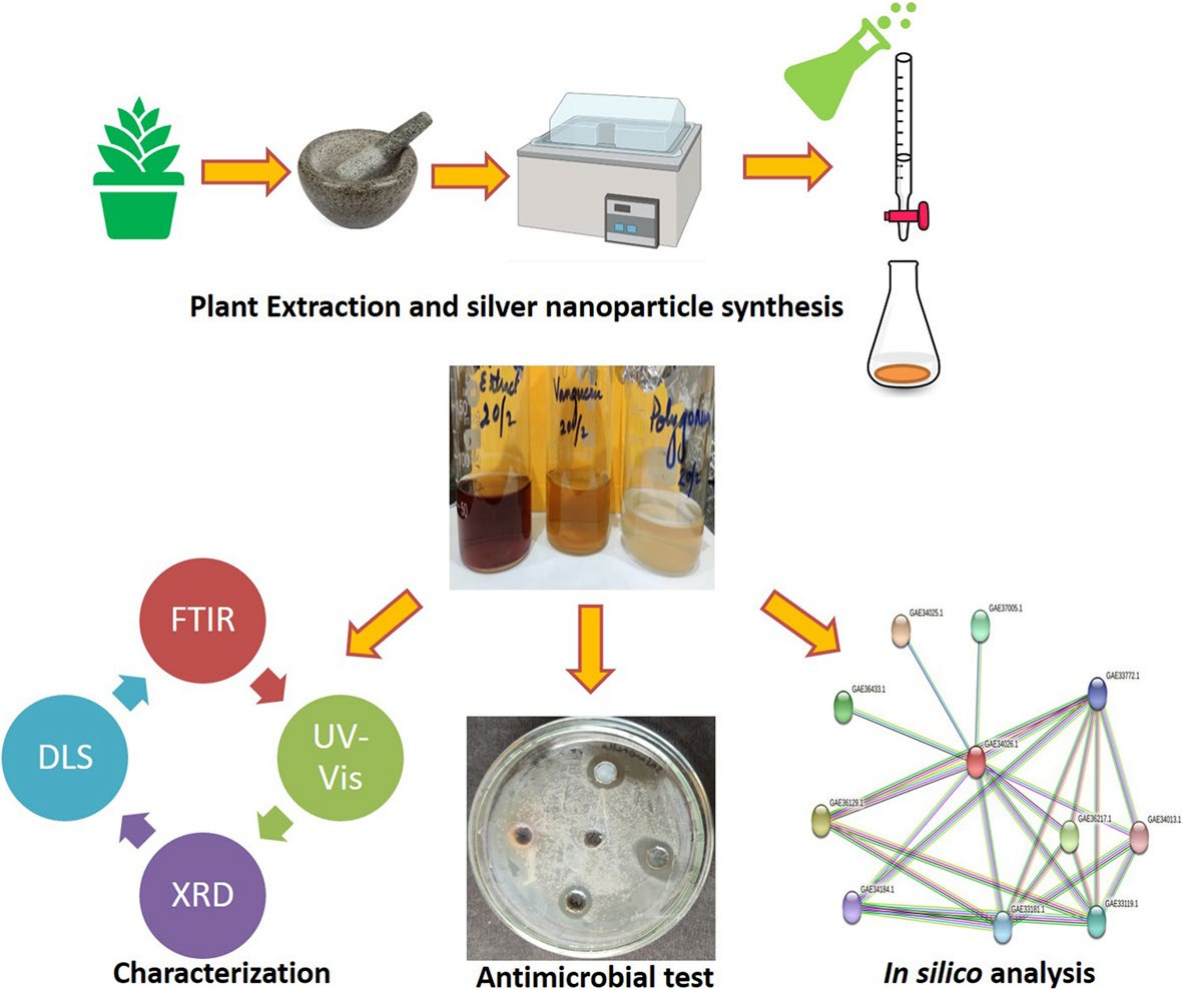

## Background

India is rich in biodiversity of several herbs, shrubs, climbers, and tree species. Ancient India was solely dependent on flora and fauna for livelihood and Ayurvedic medicines. Nowadays extensive use of such plant sources is being adopted to enhance the knowledge in agriculture, nanotechnology, pharmaceutical, and other areas due to zero side effects. Jharkhand is a very good source for indigenous flora as 40% of the total area is counted as forest and more than 160 species are having medicinal value [5]. The local tribes use many of the indigenous plants as medicine, edible species, for making dyes and ornaments [27]. The concept of ethno-medicine can also be fulfilled in Jharkhand state due to its versatility in plant sources [63, 70]. As reported by Patil et al. [58], *Madhuca longifolia* possess hepatoprotective, antioxidant, anti-inflammatory properties which is also very common in the forest of Jharkhand.

In the “zero-dimensional” world by Murthy [46], nanoparticles (NPs) are having dimensions in the nanoscale range, i.e. 1–100nm. Sizes of NPs are proportionate to the size of cells (10–100 μm), viruses (20–450 nm), proteins (5–50 nm), and DNA (2 nm broad and 10–100 nm length) [8]. Within 100-nm range, NPs are considered as one of the promising applicable materials as they play a major and commercial role in different sectors of agriculture, medicine, textile, pharmaceutical, etc. NPs have an extreme surface-to-volume ratio which dramatically changes their features in contrast to their bulk-sized materials [10, 23].

Broadly, NPs can be classified into three major classes, organic, inorganic, and polymeric. Metal/metal oxide NPs are falling within the inorganic NP class that showed huge application in different industrial sectors. Metal NPs can be used as single and in consortium with numerous surface coating materials, which can further be utilized to guide the surface properties of NPs to improve steadiness, averting NP aggregation, verifying non-noxious in physiological state and expanding a targeting activity [6, 8]. Additionally, metal NPs have an ample spectrum of antimicrobial function on innumerable Gram-positive along with Gram-negative bacteria [37, 38, 76, 78]. Metal NPs have the capacity to change the metabolic pathway of bacteria which acts as an advantage for us to suppress bacterial growth during the disease treatment [21, 81]. The fine-sized NPs also yield bactericidal outcomes which can be attained by piercing over bacterial membranes [11, 22, 23, 59, 64, 69]. Gram-positive bacteria show greater sensitivity to NPs as compared to Gram-negative bacteria [9, 37, 60, 78]. Among the several types of metal NPs namely carbon nanotubes, copper, clay, aluminium oxide, and titanium dioxide along with silver, silica is ordinarily used metal NPs that show antibacterial activity [41]. The Ag-NPs are primarily most used metal NPs, and their inhibitory effect over differing bacteria is hefty in contrast with alternative NPs [56, 59, 78]. Ag-NPs can show biocidal activity but a safer green synthesis route must be applied for this purpose.

Traditionally, mainly three methods have been used for nanoparticle synthesis. Among them, physical and chemical methods need high amount of energy, heat, and chemicals and at the end release many hazardous by-products which are dangerous for human as well as environment too. To tackle these unfavourable issues, nowadays green synthesis is the most favoured technique by the researchers. In this method, various parts of plants as well as many microbes have been used for the synthesis of nanoparticles [55]. Plant extracts that hold diverse reductants along with stabilizers have the potential to be used as an agent for the green synthesis of Ag-NPs [50, 51]. An aqueous medium, reducing agent, together with the bio-compatible stabilizing agent, is used in the green synthesis of Ag-NPs. By virtue of the occupancy of gobs biomolecules in the plant, metabolites own bio-reduction and bio-stabilization ability, and the scrutiny of those molecules urges control over the metal NP dimensions and morphology [49]. By governing the Ag-NP’s size and shape, the Ag-NP properties can be transmogrified which drives a novel green synthetic route development [57, 72].

Gram-negative bacteria are less resistant to Ag-NPs than Gram-positive bacteria because of the compositional difference in the cell wall. The peptidoglycan layer of the cell wall in the gram positive bacteria is much thicker (30nm) than gram-negative bacteria which decreases the permeability for Ag-NPs inside the cell [3]. As the Ag-NPs enter inside the cell, they release Ag+ ions, and the positive charge of Ag+ ions interacts with the negative charge on the cell wall of bacteria which leads to changes in cell wall morphology and increase in the cell permeability which disturbs the normal functioning of the cell and consequently results in cell death [67, 17]. Ag-NPs have more affinity to interact with phosphorous and sulfhydryl groups (extracellular membrane proteins) and thiol, amino groups (intracellular membrane proteins) (Abbaszadegan et al., 2015). By binding with these cellular components, Ag-NPs disrupt the structure of these proteins and ultimately affect the cell division and respiration, and result into microbial cell death [56, 68].

In this paper, three edible plants are procured from the Ranchi district of Jharkhand for green synthesis of Ag-NPs—they are *Litsea glutinosa*, *Polygonum plebeium*, and *Vangueria spinosus*. Other than these plants being edible, they also have commercial and medicinal value. *Litsea glutinosa* bark mucilages consist of hetero-polysaccharide polyuronides which are used as a binding agent [40, 62]. *Polygonum plebeium* (family *Polygonaceae*) in local is called “Chati sag, Chimti adkha, Chiti sag, Dubia Sag, Moti/Muthi saga, and Anjaban”, and small knotweed in English [53]. It is used in the treatment of pneumonia and rootstock, and seeds are cooked and eaten as a remedy for bowel complaints [74]. The third selected plant, *Vangueria spinosus*, is a synonym for *Meyna laxiflora* Robyns (family Rubiaceae) in local is called Katai sag (Roxburgh 1824). It is employed in the biliary complaints, hepatic congestion too. Its dried fruit is Narcotic, so it is utilized in dysentery [15, 29].

The aim of this paper is to identify and select the indigenous plant of Jharkhand for the synthesis and characterization of Ag-NPs to study its antibacterial activity of Ag-NPs in the inhibition of bacterial growth. Additionally, in silico study was performed to check the interactions between different antioxidant enzymatic systems that help in the survival of microorganism in stress condition. We can hypothesize that green synthesized Ag-NP bounds with these antioxidant enzymes and alter their function. It leads to the accumulation of high concentrations of free radicals that cause the death of various categories of bacteria.

## Methods

Analytic grade chemical substances were used in this experiment for the synthesis of Ag-NP synthesis and media preparation*.* Bacterial strains (*Bacillus subtilis*- NCIM2193; *Salmonella typhi*- MTCC 98) were collected from Microbiology laboratory of Department of Bioengineering and Biotechnology, BIT Mesra, Ranchi, Jharkhand. Silver nitrate (Merck & Co., Inc.), Nutrient agar (Hi-media), and Agar powder (Hi-Media) Solutions were made using the standard protocols.

### Collection of plant leaves

Three selected plants such as *Polygonum plebeium* (Chemti Saag), *Litsea glutinosa* (Maida baccus patta), and *Vangueria spinosus* (Katai Saag) (Table 1) were collected from different areas of Ranchi district of Jharkhand state, India.

**Table 1:** List of selected indigenous plant with their local name

Sl. No	Sample Name	Local Name	Sample Picture
1	*Polygonum plebeium*	Chemti Saag	
2	*Litsea glutinosa*	Maida Baccus patta	
3	*Vangueria spinosus (Meyna laxiflora)*	Katai Saag	

### Leaf extract preparation

**Fig. 1:**
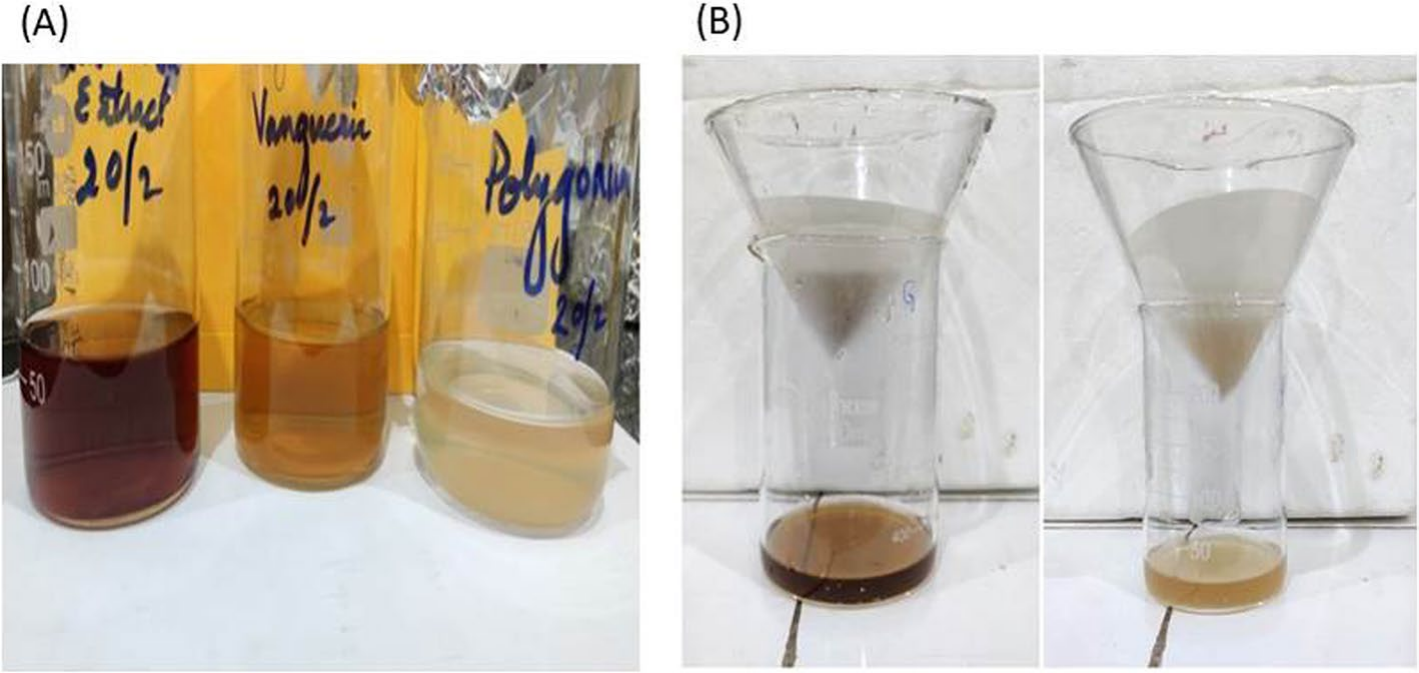
(**A**) Aqueous filtrate of *Litsea glutinosa, Vangueria spinosus*, and *Polygonum plebeium* leaves with different colour (Brown, Yellow and White). (**B**) Collection of the aqueous filtrate of *Litsea glutinosa* and *Vangueria spinosus *through Whatman filter paper into a beaker

Aqueous extracts of leaves were outturn with fresh leaves of *Polygonum plebeium*, *Litsea glutinosa*, and *Vangueria spinosus* by the following procedure: Firstly, leaves were cleaned using tap water followed by the surface cleaning using distilled water until no impurities were left on it and dried in the shady area. Then, 5 gm of dried leaf powder was weighed and added to 100 ml distilled water into a beaker. Commixture was incubated on water bath with stirring sporadically for 1h at 60°C and left to cool at room temperature (32°C) [14]. Commixture was filtered using a sieve and then Whatman filter paper of 2.5 μm pore size, and the filtrate was collected into a beaker as shown in Fig. 1. The left plant extract was preserved in the cold storage for future use.

### Synthesis

AgNO_3_ was blended in distilled water to formulate 1 and 5 mM AgNO_3_ solutions. To synthesize Ag-NPs from plant sources, AgNO_3_ was used as a precursor solution [7]. The 1 mM AgNO_3_ solutions were blended in aqueous extract of *P. plebeium*, *L. glutinosa*, and *V. spinosus* dried leaves at a ratio of 1:1, 1:2, 1:4, and 1:8 (v/v) to the volume of 10 mL in a falcon tube as shown in Table 2.

**Table 2:** List of selected plants Extract with AgNO3 in different ratio

S.No.	Sample	Plant Aqueous Extract + 1mM AgNO3	Observations
1	*Polygonum plebeium*	**1:1** – 5ml + 5ml	Brown colour solution after 24 hrs.
		**1:2** – 3.33ml + 6.66ml	
		**1:4** – 2ml + 8ml	
		**1:8** – 1.11ml + 8.89ml	
2	*Litsea glutinosa*	**1:1** – 5ml + 5ml	Brown colour solution after 24 hrs.
		**1:2** – 3.33ml + 6.66ml	
		**1:4** – 2ml + 8ml	
		**1:8** – 1.11ml + 8.89ml	
3	*Vangueria spinosus*	**1:1** – 5ml + 5ml	Brown colour solution after 24 hrs.
		**1:2** – 3.33ml + 6.66ml	
		**1:4** – 2ml + 8ml	
		**1:8** – 1.11ml + 8.89ml	

Falcon tube having *L. glutinosa* and *V. spinosus* plant extract with AgNO_3_ was again warmed up in a water bath at 100°C for 1h. After heating, the sample colour turns into greenish-brown colour. The all-falcon tube was wrapped with aluminium foil and set for an incubation period of 24h at room temperature. After 24h, the sample colour turns to yellowish-brown colour. This same procedure was repeated with 5mM of AgNO_3_ in plant extract for better results. Furthermore, the left commixture was reserved in the cold storage for an antibacterial activity test.

### Characterization

Ag-NPs were first analysed in a UV-Visible Spectrophotometer (Perkin Elmer, USA; Lambda-25) at the wavelength of 200–800nm which was equipped with “UV-Winlab” software that records and analyses the data [44]. The Ag+ ion reduction was surveilled by estimating the UV-Vis spectrum of reaction medium (sample) after blending the sample in trivial aliquant into 20 times distilled water to minimize noise present in a sample [7].

Further, the characterization of Ag-NPs was performed by various technologies such as dynamic light scattering (DLS) and particle size together with zeta potential analysis. The sample was diluted 4 times in distilled water and then analysed using DLS (Malvern Instruments, UK, Nano ZS) and zeta potential of Zetasizer Ver. 7.12 [44]. FESEM (Zeiss, Sigma 300) for Ag-NPs was also noted. FTIR (Shimadzu Corp., Japan, IR-Prestige 21) analysis was done by recording the spectra in a wavelength between 4000 and 400 cm^−1^ using a model IR-Prestige 21. For characterization in XRD (Rigaku, Japan, SmartLab 9kW), 10–15 ml of sample was dried overnight in an oven at 55–60 °C.

### Antibacterial test

Antimicrobial activity was investigated by using a well diffusion technique against Gram-positive bacteria (*B. subtilis*) and Gram-negative bacteria (*S. typhi*) [57]. Bacterial stock preparation was performed to reproduce and reinvigorate bacteria by inoculating pure culture of *B. subtilis* and *S. typhi* into 5-ml nutrient broth medium along with incubation for 24 h at 37°C [12].

All equipment, agar, and broth media were sterilized via autoclaving at 115°C and 15 psi for 30 min. Thirty milliliters of sterilized nutrient agar medium was poured in a 90-mm Petri dish; to solidify the nutrient agar solution, it was left for 15 min, followed by a 100 μL bacterial solution spread over the surface of the solid nutrient agar medium. Thenceforth, negative control (distilled water) and Ag-NP sample (5 mM of 1:2, 1:4, and 1:8 Ag-NPs) were loaded.

Briefly, in an agar plates, 100 μL sterilized tip was used to prepare the wells. One hundred-microliter samples of aqueous Ag-NP solution, aqueous extract of *P. plebeium* leaves, and distilled water were used as treatment, positive control, and negative control respectively ([57]; Rev. 1, Comment 12)**.** Next, the loaded Petri dish was incubated at 37°C for 24 h followed by quantifying the diameter of the clear zone using a ruler. Photos were shot at every interval of 24, 48, and 72 h to monitor the zone of inhibition and bacterial growth [7].

### Bioinformatics analysis

The STRING database (https://string-db.org/) was used to analyse the protein-protein interaction of glutathione peroxidase (GPx) with other enzymes involved in the antioxidant mechanism in the bacterial cell. The significantly enriched protein-protein networks were determined with the default parameter settings.

## Result and discussion

Three native and indigenous plants of Jharkhand (*Polygonum plebeium*, *Litsea*, *glutinosa* and *Vangueria spinosus*) were identified and retrieved for the biosynthesis of Ag-NPs (Table 1).

For the collection of aqueous extract for all three leaves (*P. plebeium*, *L. glutinosa*, and *V. spinosus*), first leaves were thoroughly cleaned and then boiled in distilled water. Due to the presence of metabolites, phytochemicals, and nutritional compounds, the colour change was noticed. Three different colours (brown, yellow, and white) were clearly visible (Fig. 1) for the aqueous extracts for all three plants.

These aqueous extracts were mixed with AgNO_3_ in 4 different ratios of 1:1, 1:2, 1:4, and 1:8 (v/v) to a volume of 10 mL as shown in Table 2. This same procedure was repeated with 5mM of AgNO_3_ in plant extract for better results.

The addition of 5mM of AgNO_3_ aqueous solutions in the aqueous extract of leaves at different ratios is shown in Table 2. It was observed that the aqueous extract of leaves when mixed with 5mM of AgNO_3_ aqueous solutions instantly did not show any colour change. But after a period of 24 h of incubation, the colour change was observed, yellowish and reddish to brown for all three plants *V. spinosus*, *L. glutinosa*, and *P. plebeium* respectively. The colour change in the reaction mixture (AgNO_3_ solutions + plant extract) was recorded through visual observation. Ag-NP synthesis, i.e. the reduction of Ag^+^ to Ag^0^ nanoparticles was approved by the colour change in reaction mixture solution from brown to yellow and white to (yellowish and reddish) brown upon heating and incubation period. The generation of (yellowish and reddish) brown colour is because of the surface plasmon resonance that is displayed by the nanoparticles [48]. But within a few weeks, agglomeration of Ag-NPs was observed for *V. spinosus*, so characterization steps were exempted for the Ag-NPs synthesized by this plant (UV-Vis done on both but the further characterization was done on Ag-NPs synthesized by *P. plebeium* only).

### Characterization of synthesized silver nanoparticles

**Fig. 2:**
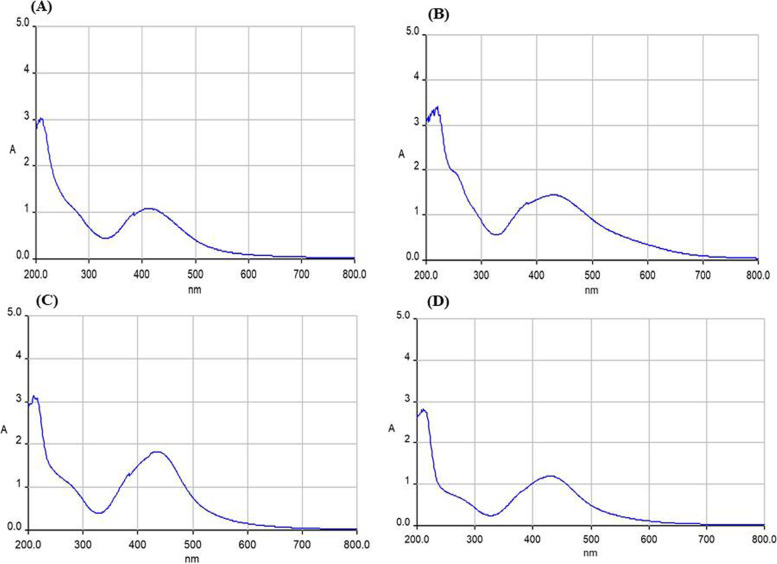
The graph obtained from the UV–Vis spectra of greener AgNPs formed from aqueous solution of AgNO3 with *Litsea glutinosa* leaf extract in different ratios-**A**) Glu 1:1, **B**) Glu 1:2, **C**) Glu 1:4 & **D**) Glu 1:8

**Fig. 3:**
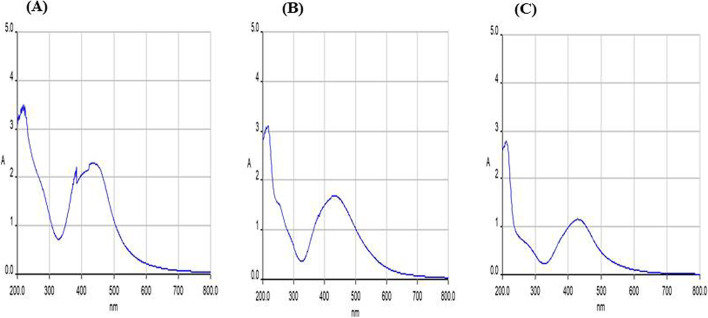
The graph obtained from the UV–Vis spectra of greener AgNPs formed from aqueous solution of AgNO_3_ with *Polygonum plebeium* leaf extract in different ratios-**A**) Poly 1:2, **B**) Poly 1:4 & **C**) Poly 1:8

The formation of green synthesized Ag-NPs using *L. glutinosa* and *P. plebeium* leaf extracts was approved by quantifying the UV-visible spectrum of the sample (reaction mixture) at 200 to 800-nm wavelengths (Figs. 2 and 3). The UV-Vis peak (20, 35, 40, and 40nm) was seen for the ratio of 1:1, 1:2, 1:4, and 1:8 of *Litsea glutinosa* (Fig. 2). For the ratio of 1:2, 1:4, and 1:8 of *P. plebeium*, the UV-Vis peak (30, 30, and 20nm) was seen in the range of 420–450nm (Fig. 3) due to contamination in the 1:1 ratio of *P. plebeium* UV–Vis was not done. Zaheer [83] proposed the presence of very specific spherical-shaped silver nanoparticles from UV-Vis peak between 410 and 450-nm range. To explore for a good result and antibacterial activity, the rest of the characterization was done on 1:4 and 1:8 of *P. plebeium*.

**Fig. 4:**
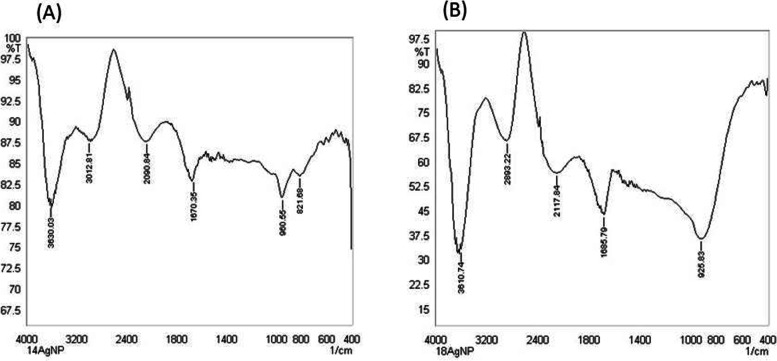
FTIR result showing graph for 2 ratios; (**A**) 1:4 AgNPs & (**B**) 1:8 AgNPs synthesized from *Polygonum plebeium* leaf extract

FTIR spectrometer was used to classify the active functional groups of the biomolecules and a phytochemical [48] present in the *P. plebeium* leaf extracts which are responsible for Ag-NP synthesis and stabilization (Fig. 4). FTIR analysis was done by recording the spectra with a wavelength range between 4000 and 400 cm^−1^ for 1:4/1:8 Ag-NP ratio [33]. For the ratio of 1:4, the absorption spectrum shows peaks at 3630.03, 3012.81, 2090.84, 1670.35, 960.55, and 821.68 cm^−1^ respectively. In 1:8 ratios, peaks were obtained at 3610.74, 2893.22, 2117.84, 1685.79, and 925.83 cm^−1^. The FTIR spectra of obtained Ag-NPs reveal diverse absorption bands ranging from 3630.03 to 821.68 cm^−1^, which approve the presence of some active functional groups with a silver [2, 73]. The different paper suggests that these functional groups, bonds, and linkages played a critical role in the stability of Ag-NPs [2, 73]. The absorption bands found in the spectra at 3630.03 and 3610.74 cm^−1^ were due to an O–H bond of alkanes, alcohols, phenols, and hydrogen-bonded carboxylic acid [16, 25, 73]. Again, the peaks at 3012.81 and 2893.22 cm^−1^ were due to the O–H or C–H vibration stretch of hydrocarbons—alkane, alkene, and aldehyde presence [16, 25]. From the study of Khadka *et al*., [28], it was revealed that the (-C=O) carbonyl group can produce a peak in the range of 1690–1630 cm^−1^ which also contributes to the bio-reduction of AgNO_3_ in the synthesis of Ag-NPs.

**Fig. 5:**
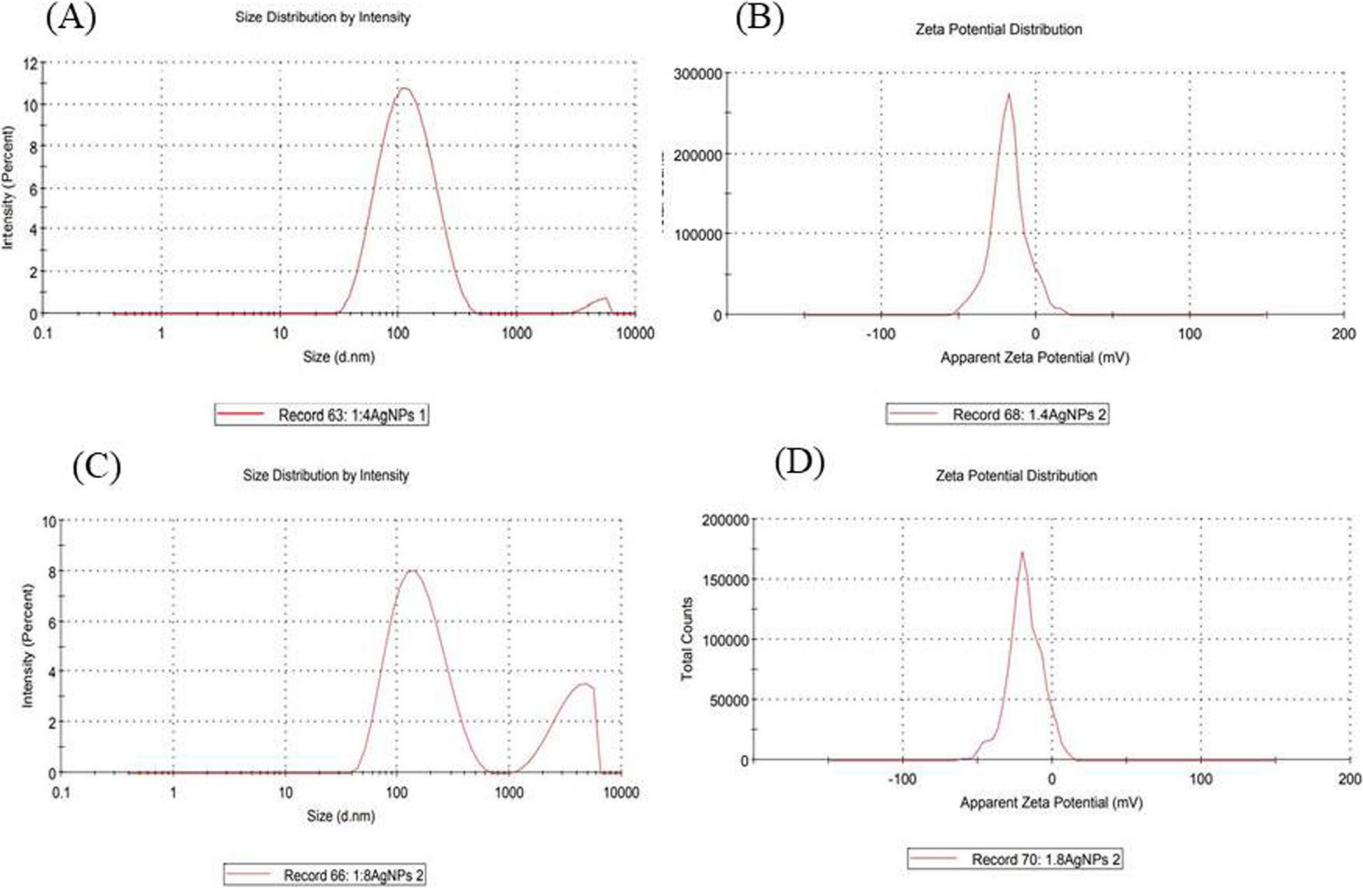
DLS analysis for (**A**) size of 1:4 AgNPs (**B**) zeta potential of 1:4 AgNPs and (**C**) Size of 1:8 AgNPs (**D**) zeta potential of 1:8 AgNPs synthesize from *Polygonum plebeium* leaf extract

DLS analysis showed the average mean particle size of synthesized Ag-NPs (1:4/1:8 ratio) has an average zeta diameter of 102.740 and 179.67nm and the average zeta potential values are −17.8 and −18.5mV (Fig. 5). The DLS analysis confirmed the negatively charged surface and good stability of Ag-NPs. The higher negative zeta potential (ξ) value has shown high dispersity of Ag-NPs and long-time stability [45, 80].

**Fig. 6:**
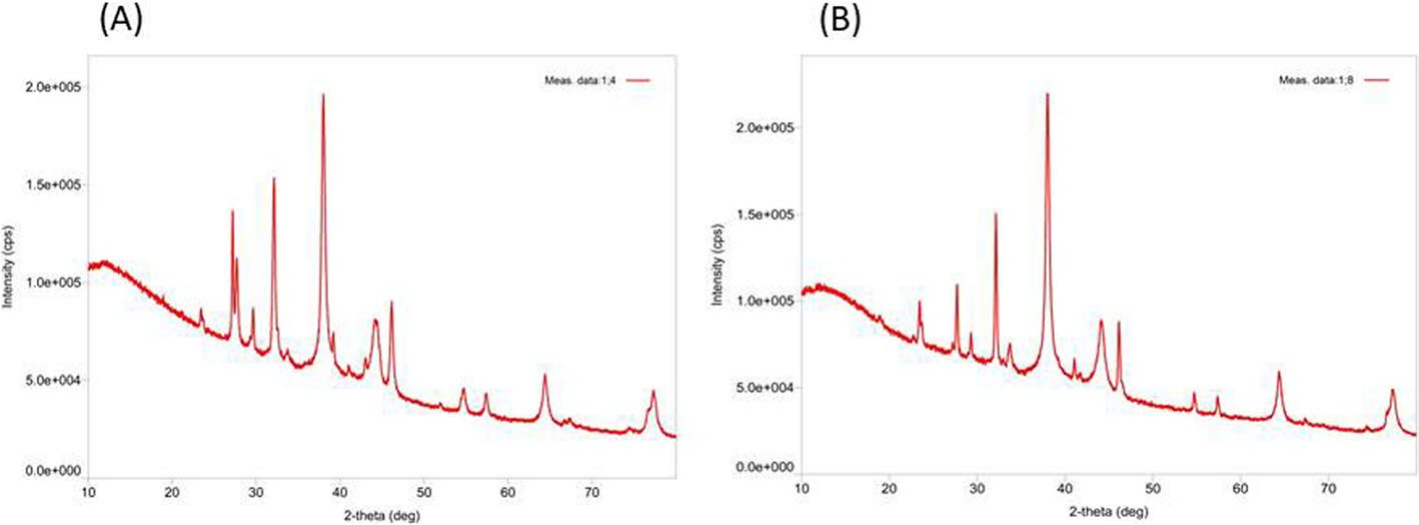
XRD pattern for (**A**) 1:4 AgNPs & (**B**) 1:8 AgNPs synthesized from *Polygonum plebeium* leaf extract

The XRD analysis confirms the crystalline nature of the NPs. The present XRD analysis (Fig. 6) confirms the presence of our synthesized Ag-NPs [48]. The XRD pattern revealed four sharp diffraction peaks at spectrum 2θ values ranging from 10 to 70 [73]. XRD pattern of the synthesized Ag-NPs produces diffraction peaks and lattice planes, which were observed at 2*θ* = 37.980, 44.280, 64.310, and 77.240 for 1:4 ratio and 2*θ* = 37.970, 44.110, 64.350, and 77.300 for 1:8 ratio [26, 28, 42, 48, 79].

**Fig. 7:**
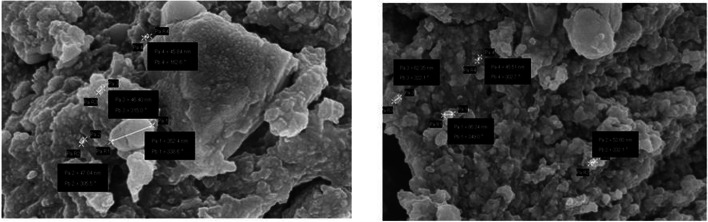
FESEM image of (**A**) 1:4 AgNPs & (**B**) 1:8 AgNPs synthesized from *Polygonum plebeium* aqueous leaf extract

Finally, for the topographic detail, FESEM was performed and the result determined the mean diameter of the surface of synthesized Ag-NPs. The micrograph was gained at various magnification using electron high tension EHT= 5.00KV, Signal A=InLens, and Magnification= 100.00KX. Result shows (Fig 7) the size of synthesized spherical Ag-NPs with an average size of 45–47 nm (45.84, 46.40, 47.04nm) and also largest particle size 352.4nm with working distance (WD) = 3.4mm for 1:4 ratio in 200nm range and 45–86 nm (62.35, 45.51, 86.34, and 52.60nm) with WD = 3.9mm for 1:8 ratio in 100nm range respectively [4, 61].

**Fig. 8:**
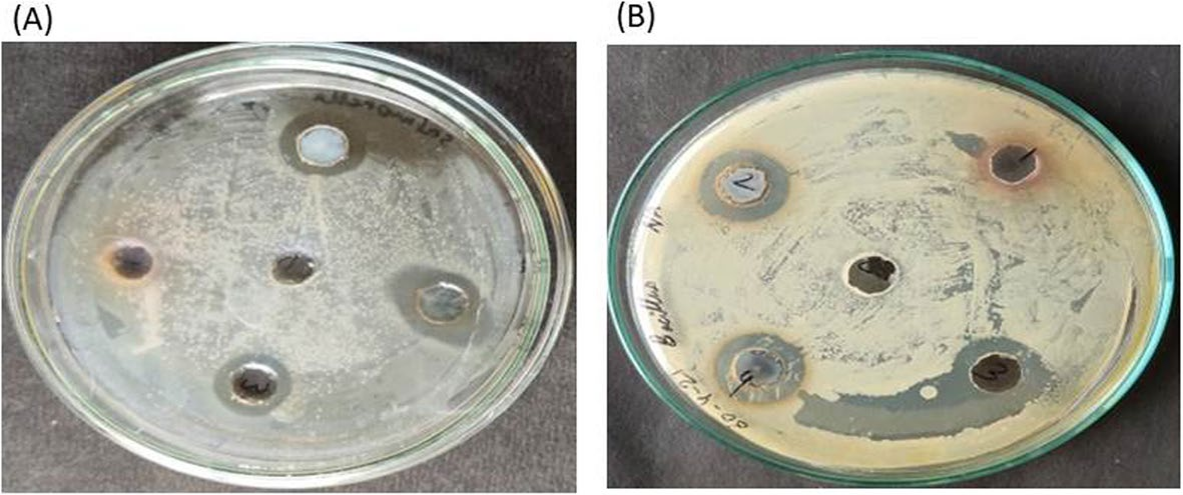
The inhibition zone occurred by *Polygonum plebeium* leaf synthesized AgNPs in bacterial culture (**A**) *Bacillus sp.* (**B**) *Salmonella typhi*

The zone of inhibition method was done to investigate the antibacterial activity. All equipment, agar, and broth media were sterilized via autoclaving at 115°C and 15 psi for 30 min. An antibacterial test was done by measuring the diameter of the inhibition zone (Table 3). In an agar plate, five wells were dug with the help of a 100-μL tip to study the interaction of aqueous extract and aqueous Ag-NP solution synthesized from *P. plebeium*. As a control, the 1st well was filled with 100 μL of autoclaved distilled water. The 2nd well was filled with 100 μL aqueous extract of *P. plebeium* leaves. The 3rd well was filled with 100 μL aqueous Ag-NP solution in the ratio 1:2. The 4th well was filled with 100 μL aqueous Ag-NP solution of ratio 1:4. Lastly, the 5th well was filled with 100 μL aqueous Ag-NP solution of ratio 1:8. After that, these plates were incubated for 24 h. The image (Fig. 8) shows the incubation period of 24 h for two bacteria *Bacillus* spp. and *Salmonella typhi*.

**Table 3:** Showing the diameters of the inhibition zone in bacterial culture

Sample	Well	***Bacillus***	***S.typhi***
Dw	Control	0 mm	0 mm
Aqueous extract	Well 1	0 mm (thin film)	0mm (thin film)
1:2 ratio AgNPs	Well 2	1.5 mm	1.4 mm
1:4 ratio AgNPs	Well 3	1.8 mm	1.7 mm
1:8 ratio AgNPs	Well 4	1.7 mm	2.2 mm

The diameter of the inhibition zone was measured as 1.5, 1.8, and 1.7 mm in *Bacillus* culture for the three ratios—1:2, 1:4, and 1:8 Ag-NPs respectively (Fig. 8A). The diameter of the inhibition zone in *S. typhi* bacterial culture was measured as 1.7, 1.4, and 2.2 mm for the three ratios—1:2, 1:4, and 1:8 Ag-NPs respectively (Fig. 8B). In both bacterial cultures, control well obtained 0 mm of diameter but well 1 was observed with the thin film of the culture surrounding a well, as it was filled with *P. plebeium* aqueous extract as shown in Table 3.

After analysing the antibacterial activity of Ag-NPs on *Bacillus* sp. and *S. typhi*, it was understandable that ratio 1:2 *P. plebeium*-synthesized Ag-NPs was less toxic to both the bacteria in comparison to 1:8 ratio *P. plebeium*-synthesized Ag-NPs. Inhibition zone of 1.7 and 2.2 mm in *Bacillus* and *S. typhi* respectively was produced by ratio 1:8 Ag-NPs whichmight be because the large amount of Ag-NPs is synthesized here. In addition, the highest inhibition zone of 2.2 mm in *S. typhi* was observed because it is a gram-negative bacteria having a thinner peptidoglycan layer compared to *Bacillus*, a gram-positive bacterium, which might perform as a defensive layer in both bacteria [19, 30, 71].

To date, exact reason for the explicit antimicrobial activity by Ag-NPs is unknown but many research papers suggest that the nano size of Ag-NPs provides a large surface area that releases enough amounts of Ag^+^ ions faster and provides good contact to microorganisms via binding to the cell membrane and piercing into the cell. After penetrating inside the bacterial cells, it disrupts the bacterial envelope. Furthermore, inside a cell, these Ag^+^ ions bind to DNA and protein with induction of ROS production, which result in denaturing the organelles and cell membrane that end up with cell lysis [13, 71, 82]. The recent work revealed that the F_O_F_1_-ATPase located in the inner membrane of mitochondria and help in synthesizing ATP for cell might perform as a responsive target for diverse metal NPs [23]. These all might be reasons for the occurrence of inhibition zone in the bacterial culture, which proves the antimicrobial and biocidal activity.

### Mechanism of antibacterial activity of silver nanoparticles

There are four different mechanisms associated with Ag-NP-induced antibacterial activity [1, 35, 47]. These four mechanisms are:

Attachment of Ag-NPs on the cell wall and membrane of bacteria
Increased rate of permeation inside bacterial cell
Modulation of signal transduction pathways
(4) Ag-NPs induced cellular level toxicity and oxidative stress

In bacterial cell, there are various kinds of antioxidant enzyme system present that help in the maintenance of the normal level of reactive oxygen species inside the bacterial cell [20, 32].

**Fig. 9:**
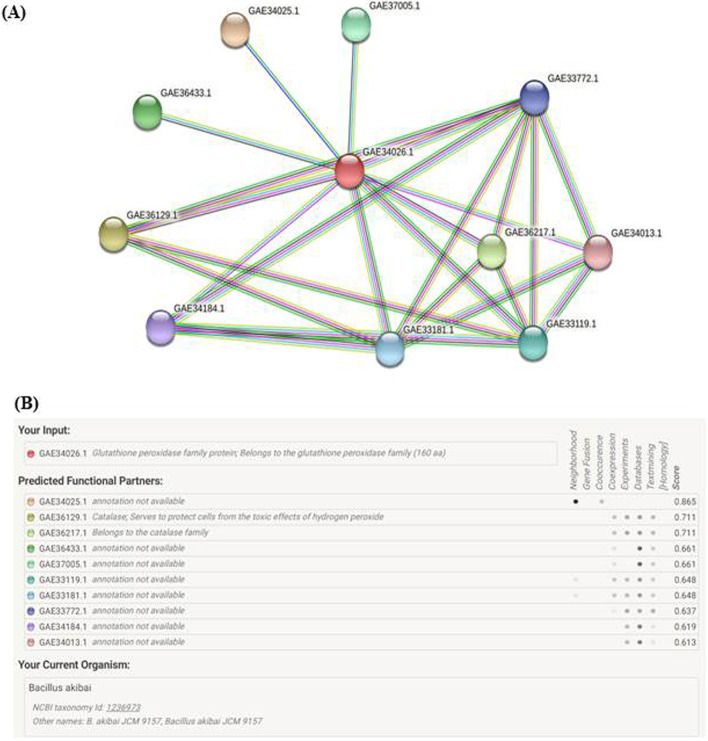
(**A**) The protein-protein interaction network of different antioxidant enzymes with **Glutathione peroxidase (GAE34026.1)** in *Bacillus akibai*. The network was created using the STRING algorithm, and strong interactions are represented by thicker lines. (**B**) Data shows the interaction score of **Glutathione peroxidase (GAE34026.1)** with other functional partners. The highest score in Neighbourhood and gene fusion is 0.865 but the annotation was unavailable. Second highest interaction score was 0.711 for two enzymes-**Catalase (GAE36129.1)** and another enzyme belongs to the catalase family **(GAE36217.1)**, both of them protects the cells from the harmful effects of hydrogen peroxide. Alteration of intracellular enzyme expressions is a key mechanism of the deterioration of Glutathione peroxidase enzyme pathway caused by Ag-NPs

The glutathione reductase enzyme is one of the antioxidant enzymatic systems that catalyses the formation of glutathione which is an important antioxidant enzyme that prevent cellular damage by oxidative stress [34, 56]. But in the case of Ag-NP-treated cell, the defense mechanism could likely be shattered due to the accumulation of elevated ROS, causing depletion of cellular glutathione leading to oxidative stress-induced cell death [31, 32, 36, 65]. The pathway of glutathione peroxidase along with interaction with the various antioxidant enzymatic systems is displayed in Fig. 9 suggesting GSH depletion could be responsible for the inactivation of other enzymatic system linked with glutathione peroxidase.

Moreover, Ag-NPs can inhibit the synthesis of proteins by denaturing ribosomes in the cytoplasm [18]. It is also reported that Ag-NPs also modulate the immune system of the human cells through inflammatory responses, which can help in controlling the population of microorganisms [75]. It was reported that Ag-NP damages the transportation of phosphate ions in *E. coli* [65].

Gram-negative bacteria are more prone to silver nanoparticles [39]. The thin cellular wall may increase the penetration of silver nanoparticles into microbial cells [39]; this suggests that the uptake of silver nanoparticles is vital for the antibacterial efficacy [52].

### Bioinformatics study

The protein-protein interaction study of glutathione peroxidase enzyme with different antioxidant enzymatic system was studied using STRING database. The target enzyme represented as GAE34026.1. Since the species of *Bacillus* strain used for antibacterial assay was not specified, so *Bacillus akibai* was chosen for the interaction study.

There are 11 nodes and 24 edges, average node degree is 4.36, average local clustering coefficient is 0.774, and ppi enrichment *p*-value is 0.000665. A small protein-protein enrichment *p*-value shows that the nodes are not random and observed number of edges is significant.

## Conclusion

As an outcome, we found green and inexpensive Ag-NP synthesis using plant extract in an aqueous environment and investigated their antibacterial activity over two bacteria. These green synthesized Ag-NPs are characterized by UV-Vis spectrophotometer, DLS, FTIR, FESEM, and XRD. Medicinal and native plants of Jharkhand namely dried leaves of *Polygonum plebeium*, *Litsea glutinosa*, and *Vangueria spinosus* were used to synthesize Ag-NPs. Ag-NP formation in the extract was observed by a colour change of *P. plebeium*, *L. glutinosa*, and *V. spinosus* extract into yellowish/reddish brown.

Due to the absence of stabilizers in the aqueous Ag-NPs synthesized from *V. spinosus* leaves, agglomeration of Ag-NPs takes place after a few weeks. On the other hand, Ag-NPs synthesized from *P. plebeium* and *L. glutinosa* leaves do not require stabilizers as an agglomeration of Ag-NPs was not formed and was stable up to 2 months without change in their properties. But for the long term, Ag-NPs synthesized from *P. plebeium* were more stable and might have prolonged antibacterial activity. The presence of the secondary metabolites in the leaves of *P. plebeium* and *L. glutinosa* might act as the reducing as well as stabilizing agent in Ag-NP synthesis.

The peak was obtained from the UV-Vis spectrometer for all 4 different ratios of (AgNO_3_ solution + aqueous extract) Ag-NPs synthesized from *P. plebeium* approving the presence of Ag-NPs. Further characterization viz., FTIR, DLS, XRD, and FESEM were done for the Ag-NP confirmation. To study the toxicity of Ag-NPs, the antibacterial test was performed on bacteria *S. typhi* and *Bacillus* sp. The finest antibacterial activity was presented by the *P. plebeium* extract-synthesized Ag-NPs of ratio 1:4 and 1:8 in *Bacillus* sp. and *Salmonella typhi* respectively.

With the in silico study, the principal antioxidant enzymatic system involved in the antioxidant mechanism of bacterial cell was analysed. From that study, it can be concluded that one of the primary reason for antibacterial effect is oxidative stress from high amounts of ROS generation. This leads to the deterioration of antioxidant enzymatic pathway of bacterial cells which result in the imbalance in normal ROS concentration inside the cell leading to the death of bacteria.

From this research work, it can be concluded that some of the native plants of Jharkhand can be used for green synthesis of Ag-NPs (within the range of 100 nm) which makes green synthesis a simple, inexpensive, and eco-friendly method. Additionally, these biologically synthesized Ag-NPs have antibacterial potential, so in the future, they can be used as an antibacterial agent in biomedical and other industrial sectors.

## Data Availability

The datasets analysed during the in silico study are available in the STRING database [https://string-db.org/].
